# Programmable Illumination and High-Speed, Multi-Wavelength, Confocal Microscopy Using a Digital Micromirror

**DOI:** 10.1371/journal.pone.0043942

**Published:** 2012-08-24

**Authors:** Franck P. Martial, Nicholas A. Hartell

**Affiliations:** Department of Cell Physiology and Pharmacology, University of Leicester, Leicester, United Kingdom; University G. D'Annunzio, Italy

## Abstract

Confocal microscopy is routinely used for high-resolution fluorescence imaging of biological specimens. Most standard confocal systems scan a laser across a specimen and collect emitted light passing through a single pinhole to produce an optical section of the sample. Sequential scanning on a point-by-point basis limits the speed of image acquisition and even the fastest commercial instruments struggle to resolve the temporal dynamics of rapid cellular events such as calcium signals. Various approaches have been introduced that increase the speed of confocal imaging. Nipkov disk microscopes, for example, use arrays of pinholes or slits on a spinning disk to achieve parallel scanning which significantly increases the speed of acquisition. Here we report the development of a microscope module that utilises a digital micromirror device as a spatial light modulator to provide programmable confocal optical sectioning with a single camera, at high spatial and axial resolution at speeds limited by the frame rate of the camera. The digital micromirror acts as a solid state Nipkov disk but with the added ability to change the pinholes size and separation and to control the light intensity on a mirror-by-mirror basis. The use of an arrangement of concave and convex mirrors in the emission pathway instead of lenses overcomes the astigmatism inherent with DMD devices, increases light collection efficiency and ensures image collection is achromatic so that images are perfectly aligned at different wavelengths. Combined with non-laser light sources, this allows low cost, high-speed, multi-wavelength image acquisition without the need for complex wavelength-dependent image alignment. The micromirror can also be used for programmable illumination allowing spatially defined photoactivation of fluorescent proteins. We demonstrate the use of this system for high-speed calcium imaging using both a single wavelength calcium indicator and a genetically encoded, ratiometric, calcium sensor.

## Introduction

Confocal laser-scanning microscopes (CLSM) are widely used to obtain high-resolution images of biological specimens. In conventional fluorescence microscopy, more than 90% of the observed fluorescence may originate from parts of the sample that are out of focus and this scattered light from planes above and below the area-of-interest obscures the detail of the specimen within the desired plane of focus [1]. Confocal microscopes overcome this problem by using a pinhole located in a conjugate image plane to reject light that originates outside the focal plane of the object. This optical sectioning improves the apparent spatial resolution of the image. Conventional confocal microscopes generate an image of the sample by scanning a laser light source over the specimen with a pair of galvanometer driven mirrors and collecting the light that passes through the pinhole. Sequential scanning on a point-by-point basis limits the speed of image acquisition; even the fastest commercial instruments struggle to resolve the temporal dynamics of the fastest cellular events.

Several alternative approaches have been used to enhance the acquisition speed of confocal microscopes. High-speed resonant galvanometers scan at faster rates than conventional galvanometers but these must still scan an entire image, point by point. The increase in speed is gained at the expense of the single pixel dwell time and this leads to an inevitable reduction in image quality. Line or slit scanning systems have been developed that produce quite significant increases in speed [2]. Slit-scanning systems employ a cylindrical lens to expand a laser beam into a line that is then rapidly scanned across the specimen on a line-by-line basis. The increase in acquisition speed is, however, countered by reduced axial resolution because a slit aperture is used in place of a pinhole and this decreases the degree of optical sectioning.

An alternative approach to faster scanning that suffers neither from a reduced image quality or reduced optical sectioning is to scan multiple beams in parallel and use an array of pinholes and detectors to collect the image. Scanning disk systems such as the Nipkov disk are based on a spinning mask of pinholes that can simultaneously illuminate many discrete points [3]. When the disk spins at high-speed, laser light passes through the pinholes and illuminates the whole specimen almost simultaneously. A camera is used to collect the light that passes back through the pinholes. Optical sectioning occurs because light emitted above or below the focal plane does not return through the array of pinholes. A major disadvantage of scanning disk systems is that the excitation pathway suffers from very low efficiency. The disc rejects the vast majority of the illumination light because the spacing between neighbouring pinholes must be large to maintain a reasonable confocal effect. The introduction of more and more powerful lasers overcomes this problem but at a considerable financial cost, particularly if multi-wavelength imaging is required. Nipkov disks have other significant limitations. First, they have fixed pinhole diameters and spacing. These two factors determine the level of optical sectioning and they can only be optimised for one particular numerical aperture of the objective lens. Changing the degree of optical sectioning requires physically removing and replacing the disk, which is not possible in most systems and troublesome in others, requiring re-alignment. The introduction of microlenses that enhance the transmission of excitation light and electron magnifying (EM) CCD cameras which improve detection efficiency have improved the utility of these systems but the quality of results obtained from these systems depends entirely on the brightness of the specimen and so their use depends on the particular application.

More recently, non-scanning techniques using programmable array devices such as transmissive or reflective liquid crystal displays (LCDs) and digital micromirror device (DMD) arrays have been used to produce optical sectioning [4], [5], [6], [7], [8], [9], [10], [11], [12]. These devices permit optical sectioning because they act as both spatial light modulators that can define patterns of illumination and/or pinholes that allow detection of light from the image plane. A significant advantage of systems based on programmable array devices is that they can be easily configured to perform wide-field, confocal and standard fluorescence microscopy, and allow easy adjustment of the pinhole size and separation.

Although several examples of DMD based optically sectioning microscopes have been published, none of these have been demonstrated to operate in full frame mode at speeds high enough to capture fast biological events. High-speed point detection has been achieved using a DMD in combination with an acousto-optic deflector [8], [9], which is used to position the excitation light so as to overcome the inefficiency of point illumination with a DMD. Fluorescence speckle microscopy has also been implemented with a DMD but acquisition of confocal images requires subtraction of images formed either sequentially on the same camera or simultaneously on two different cameras. Even with the fastest control software available and using two cameras, this mode is only likely to achieve a maximum acquisition rate of 10 frames per second [10]. A liquid crystal optically sectioning microscope has been reported that can operate at speeds up to 20 fps but this also requires two, high sensitivity cameras to obtain a confocal image [12].

Here we report the development of an optically sectioning microscope that utilises a DMD as a spatial light modulator to provide programmable confocal optical sectioning with a single camera, at high spatial and axial resolution at frame rates limited by the speed of the camera. The DMD acts as a solid state Nipkov disk but with the added ability to change the pinholes size and separation and to control the light intensity on a mirror-by-mirror basis. The use of only mirrors in the emission pathway of the confocal module increases light collection efficiency and ensures image collection is achromatic. Combined with non-laser light sources, this allows low cost, high-speed, multi-wavelength image acquisition without the need for complex wavelength-dependent image alignment. Software control is via a USB connection allowing simple incorporation into existing a fluorescence imaging systems. The DMD can also be used for programmable illumination and so the system can be used for caged release, fluorescent protein activation or fluorescence recovery after photobleaching (FRAP) measurements.

## Materials and Methods

**Figure 1:**
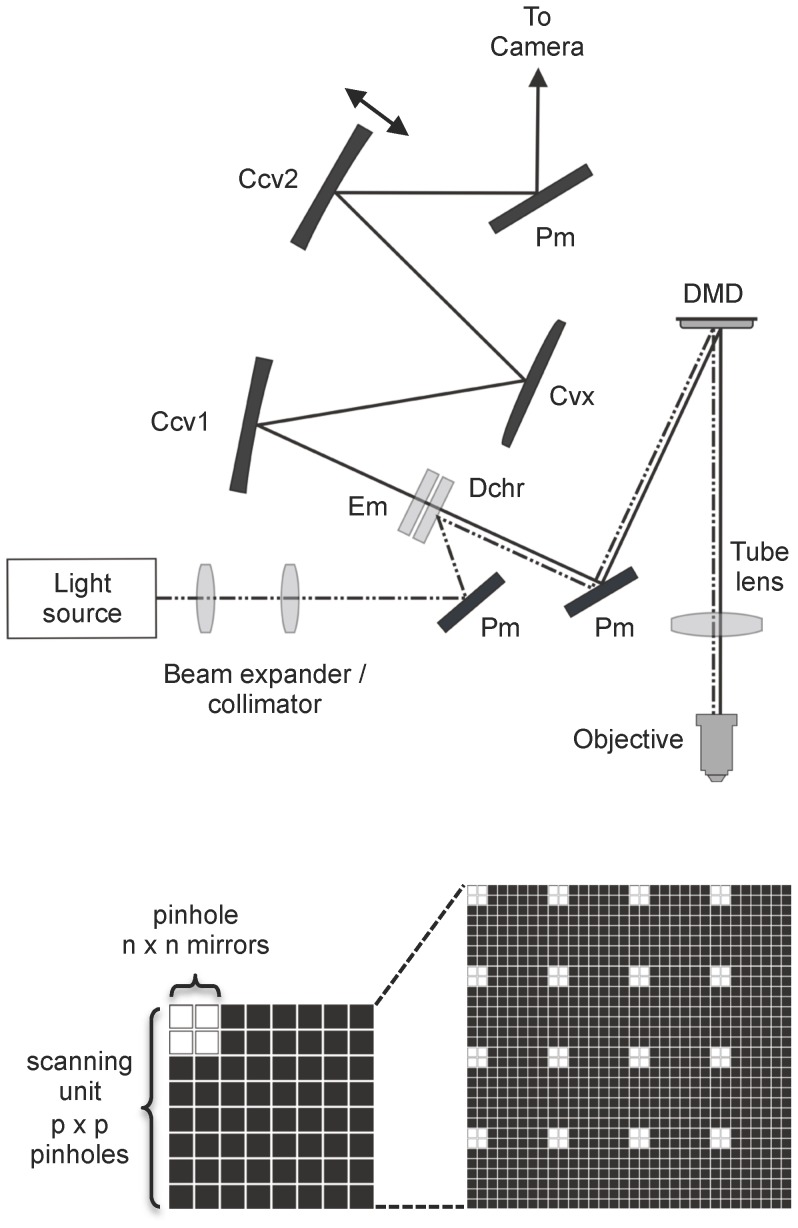
Schematic diagrams of the DMD confocal optical pathway and the mirror arrangement for pinhole formation and scanning. A. Light from the excitation source was expanded and collimated and sent to the DMD via a dichroic mirror. Mirrors in the “on” position on the DMD send the excitation light through a tube lens to the microscope objective and the specimen. Light emitted from the specimen travels back along the same path but it then passes through the dichroic mirror (Dchr). An emission filter (Em) ensures that only the desired excitation wavelength can reach the camera. A modified Offner Triplet arrangement consisting of three separate curved mirrors (CCv1 and CCv2 concave mirrors, r = 207; Cvx convex mirror, r = 103.5) and a single plain mirror (Pm) were then used to form an image on the CCD of an EM camera positioned at a second conjugate image plane. The double-headed arrow next to CCv2 illustrates the approximate plane of movement that allows a change in the magnification of this optical relay. B. A scanning unit consists of n×n micro mirrors in the “on” position, acting as a pinhole, in a square of p×p pinholes. Full scan of the unit is obtained by presenting a series of p2 scanning units with the pinhole in different positions. Different values of n and p define different configurations, referred to as “n×p”, and give different levels of confocality. The figure illustrates a 2×4 confocal configuration.

The DMD confocal device was built on a Nikon Eclipse FN1 upright microscope using either a 10× (NA 0.3), 40× (NA 0.8) or 60× (NA 1.0) water immersion objective. A schematic diagram of the optical layout is shown in Figure 1A. A DMD (DLP discovery 3000 board; Texas Instruments, Dallas, TX) fitted with a high-speed ALP-3 controller board (Vialux, Chemnitz, Germany) was positioned at the primary conjugate image plane. The DMD consisted of an array of 1024 by 768 mirrors (SVGA) with a pitch of 13.68 µm. Each mirror can be independently tilted by ±12° along its diagonal axis between two different resting positions. The DMD was rotated by 45 degrees to keep the deflection of the mirrors in the horizontal plane.

Several types of light source were evaluated. Initially, an argon-ion laser (Stellar-Pro, Modu-Laser, USA) was used. The excitation wavelength was selected with a suitable notch filter. The beam was expanded through a telescope to a diameter of approximately 11 mm and reflected via a dichroic mirror to illuminate a central area of approximately 600×600 DMD mirrors at an angle of 24 degrees. Other light sources evaluated were a mercury lamp fitted with a liquid light guide (Intensilight, Nikon, UK) and a LED based light engine (Aura, Lumencor, USA). The outputs from each device were collimated to illuminate the central area of the DMD. For the mercury lamp, band pass filters (Semrock, USA) were used to obtain the appropriate excitation wavelength. Light from each of the excitation sources tested was reflected via a plain mirror (Pm) onto a dichroic mirror (Dchr), to a second plain mirror and onto the DMD. Light reflected from the DMD passed through the tube lens of the microscope (f = 200 mm) to the microscope objective where it was focussed onto the specimen. Since the excitation beam has to pass through the tube lens after reflecting off the DMD, we found that the best quality images were obtained if the beam was made to diverge slightly so that the tube lens acted to collimate the beam onto the back aperture of the objective.

Light emitted from the specimen travels back along the same path and transmits through the dichroic mirror and an emission filter. A modified Offner triplet arrangement consisting of three separate curved mirrors (Ccv1 & Ccv2 - concave mirrors, r = 207; Cvx - convex mirror, r = 103.5) and a pair of flat mirrors were used to form an image on the CCD of an electron-multiplying camera positioned at a second conjugate image plane. The modified Offner triplet has four principal advantages. First, this arrangement allows an image of the DMD to be formed that is normal to the plane of the DMD, which avoids astigmatism caused by tilting of the wavefront of light reflecting from the DMD, relative to the optical axis. Second, the high reflectivity of mirrors allows a much more efficient transfer of light than would be possible with a lens based system. Third, the mirror arrangement is achromatic ensuring that images are aligned regardless of emission wavelength. Fourth, by using physically separate concave mirrors on the outer radius of the optical path, the Offner arrangement can be modified by altering the position of the last concave mirror (Ccv2), which allows a small change in magnification. Increasing the magnification by a factor of 1.17 allowed the pitch of the DMD mirrors to be aligned precisely with the pixels of the CCD camera and this optimises image resolution and collection efficiency. Images collected by the electron-multiplying CCD camera (Hamamatsu C9100-13) were transferred to a PC for recording and analysis with Igor Pro Software (Wavemetric Inc, Oregon USA).

### DMD Scan Control

Equation 1 was used to calculate the airy disk diameter (Φ) projected on the DMD for diffraction-limited spots focused on the specimen. With a DMD mirror pitch of 13.68 µm the closest achievable mirror arrangements to form pinholes optimized for 1 airy disk were 2×2 mirrors for ×10 and ×20 objectives and 3×3 mirrors for ×40 and ×60 objectives. M is the total magnification from objective to conjugate image plane, λ = the wavelength of light and NA is the numerical aperture of the objective lens.



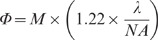



The ALP-3 high-speed controller simplifies control of the DMD. The controller provides 1 Gbit of memory into which sequences of binary or 8 bit frames can be loaded via a USB connection. The device can display binary frames at a rate of 16300 per second, which represents an interval of about 60 µs. Any single mirror can alternate between one of two resting states; those that project light onto the specimen are designated as being in the “on” position whereas the others are in the “off” position. If frames are sent in 8-bit format, the mirrors oscillate between on and off states at a frequency defined by the bit level. This allows the light intensity to be adjusted on a pixel-by-pixel basis. Conventional wide-field fluorescence illumination can be realised by setting all mirrors to the “on” position and the illumination intensity can be controlled programmatically by changing the proportion of time that the mirrors are in the on or off position. Sequence generation, loading and display were controlled using custom procedures written for Igor Pro.

### Programmable illumination for confocal sectioning

Confocal imaging was achieved by defining sequences of moving “pinholes” which represent patterns of mirrors in the on position separated by mirrors in the off position. In the on position, light is directed to the sample. On mirrors behave like pinholes in a conventional confocal microscope because only light emitted from the plane of focus should return along the same path and be directed to the camera. Out of focus light will hit a neighbouring mirror and will be rejected if it is in the off position. The degree of optical sectioning will depend upon the size and separation of the pinholes. To scan an entire field of view, a series of DMD frames were programmed to form arrays of identical scanning units. Each scanning unit consisted of a square array of p×p micromirrors within which a sub array of n×n mirrors, acting as a pinhole, will be in the on position at any given time (figure 1B). The pinhole is moved sequentially to a different position in the scanning unit until the entire area of the scanning unit has been turned on. Each scanning unit operates in parallel. Scan speed and the degree of optical sectioning are determined by the size of the pinhole and the width of the scanning unit, which determines the pinhole spacing. For a square scan unit, the number of DMD frames required to completely scan the entire field of view is determined by the scan unit width (p) divided by the pinhole width (n). Therefore, for a pinhole of 2×2 mirrors and a scan unit of 8×8 pinholes (i.e. 2×8 = 16 mirrors), the number of frames required is (16/2)^2^ = 64 frames. Since the speed of DMD frame update is around 60 us, the theoretical maximum duration over which all these DMD frames could be presented is 64*60 us = 3.8 ms. A complete scan can be repeated 260 times per second which far exceeds the full frame maximum speed of the camera. To minimize edge effects on images, the entire sequence of frames can be repeated after shifting the pinhole by n/2 mirrors.

The effects of pinhole size and pinhole separation on the degree of optical sectioning were tested. Series of images of 4 or 6 µm diameter fluorescent beads (Life Technologies, Carlsbad, California) were taken in the axial plane (Z-sections) at 0.5 µm intervals and fluorescence intensity profiles of the beads were determined. The data were fitted with a Gaussian function from which the axial height of the beads was estimated.

Sample images were taken of marrow pollen (*cucurbita*) and from pond snail neurons *(Lymanaea stagnalis*) filled with Alexa Fluor 568. A dual band filter set was used (Chroma 59022) in combination with a light engine (Lumencor), which allows fast switching of excitation ranges of 450–490 nm and 550–590 nm. Emission ranges between 500–540 and 600–670 nm were collected. Series of wide-field and confocal images using a range of pinhole configurations were collected in the axial plane and maximum projections of the Z-stacks created. For comparisons with a commercial CLSM, a Leica SP2 microscope equipped with an argon-ion laser was used. Z stacks were collected using an objective with an equivalent N.A and with an optical zoom set to match the overall magnification of the DMD microscope. Line averaging of 4 was used and maximum projections were created as above.

For live cell imaging of Purkinje neurons parasagittal slices were prepared from the cerebellar vermis of 12–21 day old male, Wistar rats. Animals were deeply anesthetized with isoflurane and decapitated. Cerebella were rapidly removed and placed in a chilled (<4°C), extracellular solution of the following composition (in mM): 250 sucrose, 2.5 KCl, 2 CaCl_2_, 1 MgCl_2_, 1.25 NaH_2_PO_4_, 10 D-glucose, gassed with 95% O_2_ – 5% CO_2_, pH 7.4. Slices were cut to a thickness of 150–200 µm and then transferred to a holding chamber for incubation prior to recording. A standard artificial cerebrospinal fluid (aCSF) comprising (in mM) 126 NaCl, 3 KCl, 2.5 CaCl_2_, 1.3 MgSO_4_, 1.25 NaH_2_PO_4_, 26 NaHCO_3_, 10 D-glucose gassed with 95% O_2_ – 5% CO_2_, pH 7.4 was used for incubation and recording. For electrophysiological recording, a cesium based solution was used that contained (in mM): 145 cesium methanesulphonate, 10 QX-314, 2 MgCl_2_, 5 K_2_ATP, and 5 N-2-hydroxyethylpiperazine-N′-2-ethanesulfonic acid (HEPES), pH 7.3, osmolarity adjusted to 280 mOsm/l. Oregon Green BAPTA-1 (Life Technologies, Carlsbad, California) was added to a final concentration of 250 µM. Whole cell recordings were made using an Axopatch 200B amplifier (Molecular Devices, Sunnyvale, CA) and a National Instruments X-series data acquisition board. Procedures written in Igor Pro enabled simultaneous control of image collection and electrophysiological recording.

Ratiometric calcium measurements were made from hippocampal neurons expressing GCaMP2-mCherry. This genetically encoded sensor was constructed by fusing mCherry [13] to the C-terminus of GCaMP2 [14] within the pcDNA3.1B vector (Invitrogen). Neurons were prepared from the hippocampi of p1 rat pups according to standard procedures and transfected with Lipofectamine2000 (Life Technologies, Carlsbad, California) after 7–8 days in culture. Imaging was performed at least 7 days later. High-speed ratiometric imaging was achieved by collecting bursts of 360 images at 20 ms intervals whilst alternating the excitation wavelengths between 470±20 nm and 570±20 nm. Excitation wavelength was controlled by TTL outputs that were synchronized with image capture. Images at each wavelength were collected as an interleaved tiff stack and separated after collection for subsequent analysis. Image capture was also synchronized to the DMD. Camera pixel binning by up to 8×8 was used for fast imaging and the image exposure was set to correspond to the time required to run a complete sequence of DMD frames required to scan a complete confocal image. Data shown represent rates of 50 frames per second, equivalent to 25 ratio image pairs per second. Neurons were stimulated electrically with 1 ms pulses with a pair of platinum wires placed either side of the neuron. Analysis was performed offline using Igor Pro.

pRSETa mEOS2 (Adgene, Cambridge, MA) was grown in a DH5α bacterial strain and colonies were spread onto a glass slide with a coverslip over the top to produce a “carpet” of fluorescent bacteria. mEOS2 fluoresces green when excited with blue light but displays only weak red fluorescence when excited with green/yellow light [15]. Following excitation at 405 nm, the fluorophore is photoactivated leading to a decrease in green fluorescence and increase in red fluorescence. A violet LED light source was used to excite the bacteria with a programmed pattern of light projected onto the specimen. Images of green and red fluorescence were taken after a 60 second exposure period.

## Results

### Characterisation of axial resolution

**Figure 2:**
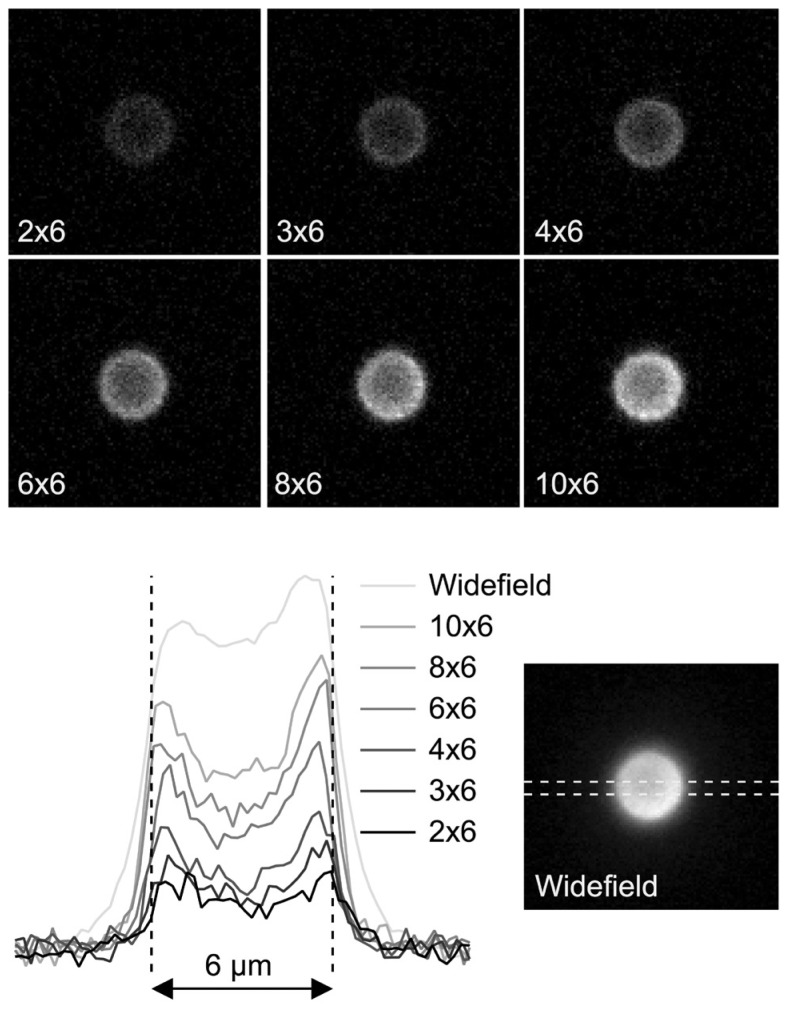
The effect of pinhole size on image contrast and resolution. Images were taken through the axial centre of 6 um beads using pinhole sizes ranging from 22 mirrors to 102 mirrors. A constant proportional separation was used such that each pinhole was separated in x and y dimensions by a distance of 6 pinholes. Line profile measurements through the centre of the beads for each configuration are plotted below. The dashed horizontal lines illustrate the position from where the line profile measurements were taken.

**Figure 3:**
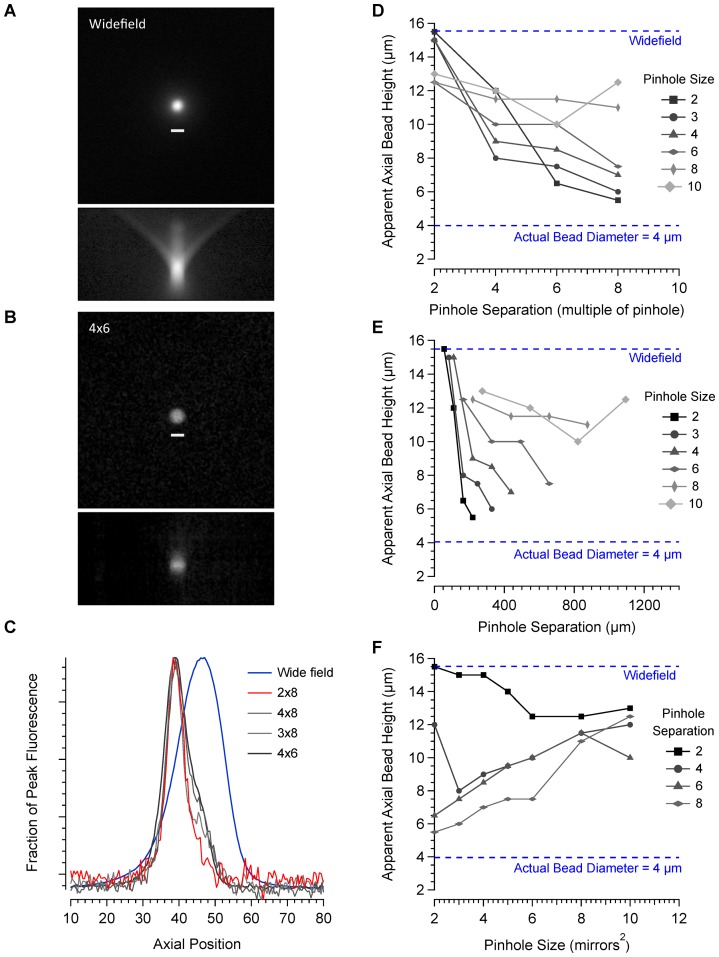
Characterisation of axial resolution. A. Maximum projection of wide-field images of a 4 µm bead taken over an axial distance of 120 µm at 0.5 µm intervals. Below is an axial projection from a frontal plane. B. Images taken of the same bead using a 4×6 pinhole configuration and processed in the same way. Axial projections were made for a range of different pinhole configurations and line profiles drawn through the centre of the bead. C. Axial line profiles comparing 4 different pinhole configurations with images taken in wide-field are shown. Data were fitted with a Gaussian curve to estimate the axial height of the bead. Figures D–F illustrate the relationship between axial resolution, pinhole size and pinhole separation.

Fluorescent beads were used to assess the effect of varying pinhole size and separation on the spatial (x and y) and axial (z) resolution of the DMD microscope (See Figure 1 for schematic diagram). The results are summarised in figures 2 and 3. Figure 2 illustrates the effect of increasing pinhole size at a fixed relative separation (i.e. a fixed multiple of pinhole width). With 6 um hollow beads an outer ring could be resolved from a less bright central region. Line profiles were drawn through the bead to illustrate how pinhole size affected brightness and contrast in the x and y planes. Increasing pinhole size led to an increase in signal, however image quality declined with pinhole sizes greater than 6^2^ mirrors because noticeable pixelation of the images occurred. The greatest contrast was obtained with a pinhole configuration of 4×6 as assessed by measuring the ratio of the fluorescence of the outer ring compared to the inner centre.

For a 60 times magnification objective with a numerical aperture of 1, the theoretical pinhole size required to produce the best optical sectioning is approximately 3^2^ mirrors. At this pinhole size, axial resolution improved considerably compared to wide-field mode as the pinhole separation increased up to the maximum separation measured of 8 pinhole widths. Smaller pinhole sizes produced a small improvement in axial resolution but the signal to noise ratio decreased leading to reduced image contrast. Larger pinholes produced a better signal to noise ratio, better contrast and good axial resolution provided the separation between pinholes was sufficient to prevent cross talk. With pinhole sizes up to 8^2^ mirrors, the best optical sectioning was obtained at a separation of 8 pinhole widths. Plotting pinhole diameter against absolute pinhole separation revealed that a distance of at least 160 µm between pinholes, regardless of size, is essential for a good degree of axial sectioning. For a given relative separation, axial resolution declined as pinhole size increased. These data confirm that both pinhole size and separation are important for determining the level of confocality.

**Figure 4:**
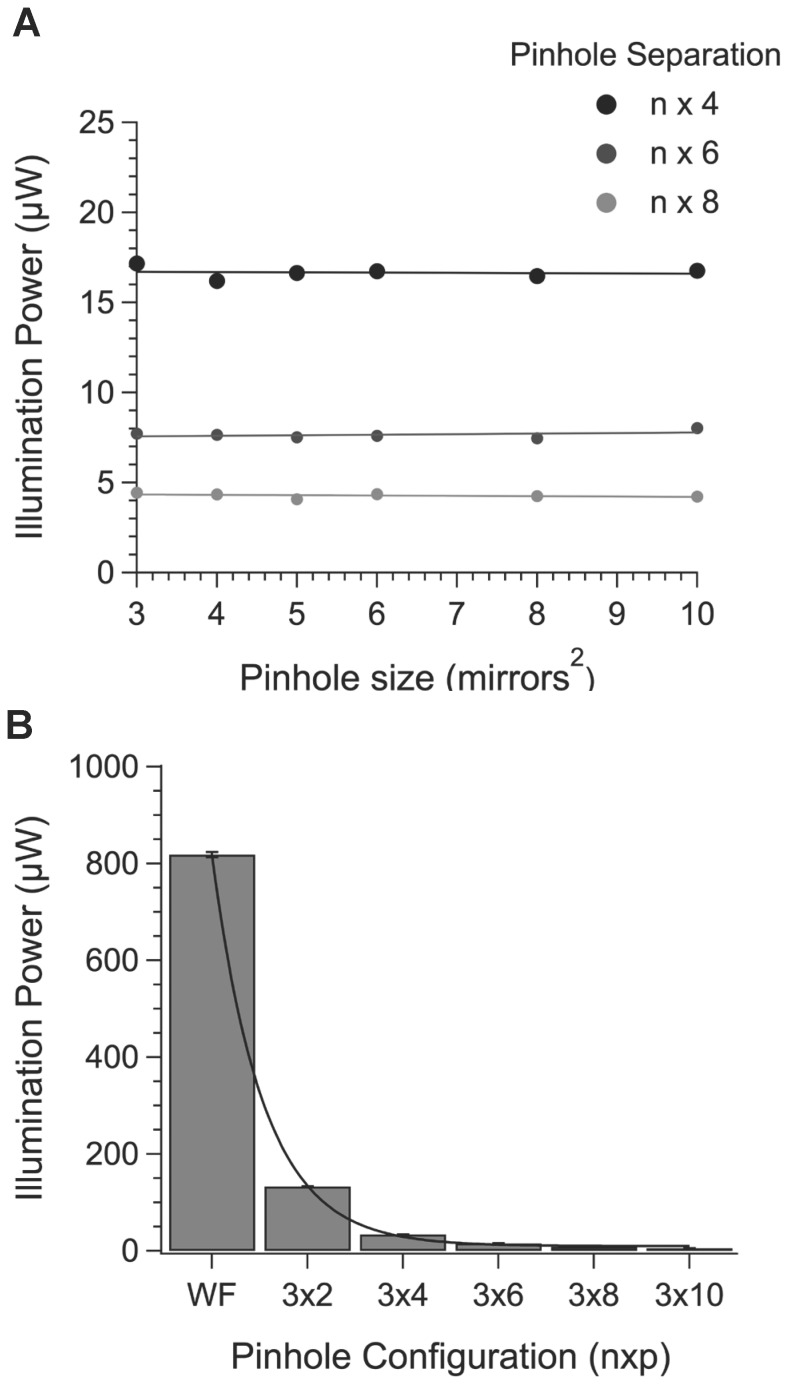
The relationship between pinhole size and separation on output power measured at the specimen. A. The average power at the specimen plane was measured for each pinhole configuration. Separations of 4, 6 and 8 pinhole widths are shown. B. The average power measured at the specimen plane for a 32 pinhole at different separations is shown. Superimposed is an exponential fit of the data.

**Figure 5:**
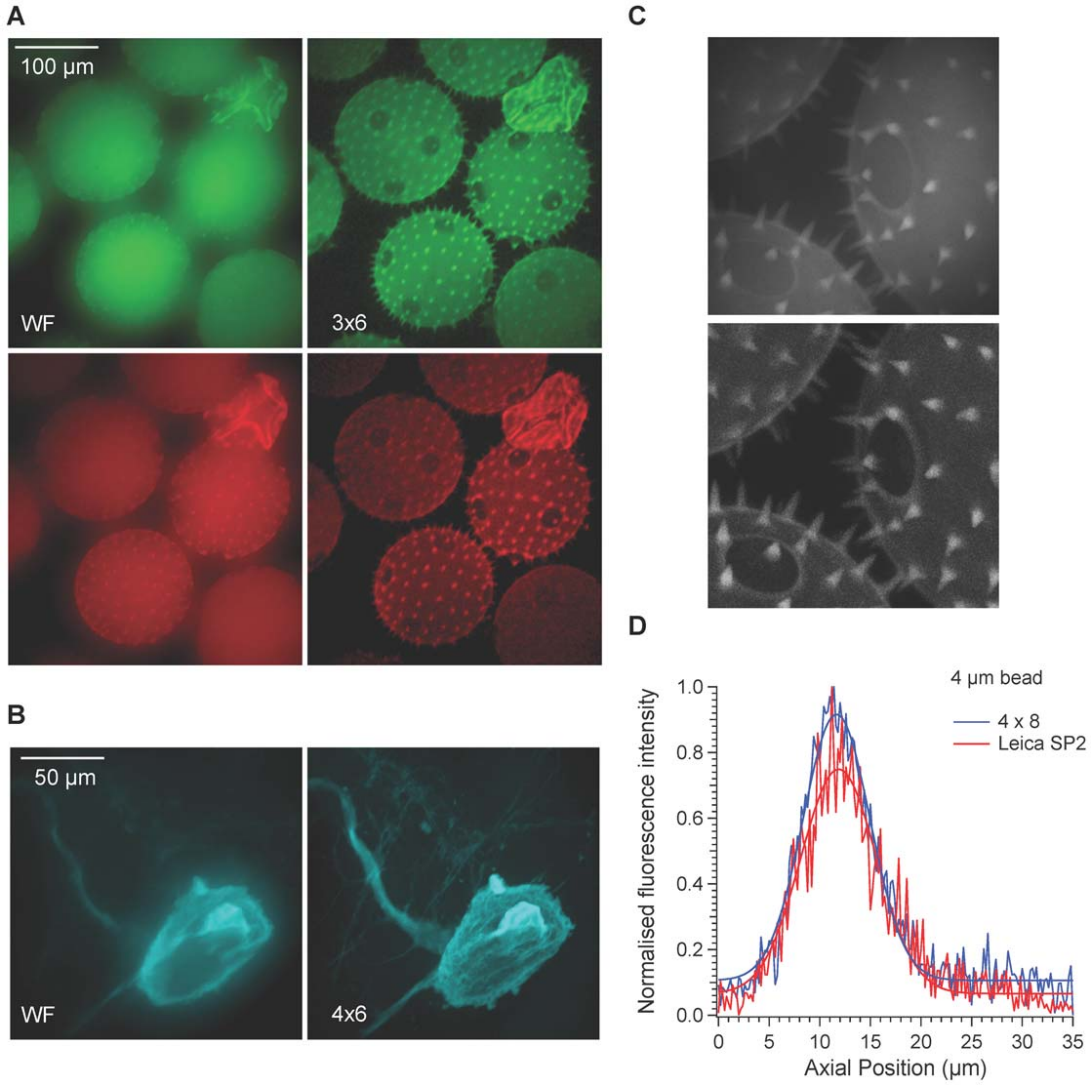
Examples of wide-field and confocal images of biological specimens. A. Wide-field (left) and confocal images (right) of cucurbita pollen grains. Excitation wavelengths were 470±nm and 560±20 nm and emission wavelengths of 500–540 nm and 600–670 nm were collected. Images represent maximum projections of stacks of images taken in the axial plane. B. Wide-field and confocal images of a snail neuron filled with Alexa Fluor 568. A cyan look up table was used for clarity. C. A comparison of cucurbita maximum projections captured with the DMD confocal (top) and Leica SP2 CLSM (bottom) with 60 and 63× objectives with N.A of 1 and 0.9 respectively D. Axial profiles of 4 µm beads measured with the DMD confocal with a 4×8 pinhole configuration and Leica SP2 CLSM with an optimal pinhole setting of 1 airy unit.

Separations less than 160 µm limit axial resolution through cross talk between pinholes. Larger separations increased the degree of axial sectioning but with a reduction in the signal to noise ratio. The explanation for this is shown in Figure 4, which illustrates the relationship between pinhole size and separation and excitation light power measured at the specimen. Over a complete cycle of mirror positions required to produce an image, pinhole diameter has no effect on the amount of light transmission provided the pinhole separation is expressed as a proportion of the pinhole size (Figure 4A). For a given pinhole size increasing the pinhole separation leads to an exponential decline in excitation power because the fraction of the total number of mirrors in the on position at any one time decreases exponentially (Figure 5B). Wider separations lead to a significant drop in excitation power and the associated reduction in the signal to noise ratio eventually precludes any theoretical improvement in axial resolution. This inefficiency is unavoidable with all Nipkov disk type microscopes but further improvements in axial resolution should be easily achievable by increasing the power of the excitation source.

We next assessed the image quality of the microscope using a range of biological specimens. Figure 5A compares wide-field and confocal images of the marrow pollen *cucurbita* using a 20 times objective at excitation wavelengths of 470±20 nm and 570±20 nm. Emission wavelengths of 500–540 nm and 600–670 nm were collected. The improved contrast and resolution apparent from confocal imaging is apparent. One of the benefits of the mirror based system used to relay the image formed on the DMD to the camera is that it is achromatic. Images collected at different wavelengths are therefore automatically aligned perfectly. The microscope was also able to detect fine detail as shown with the wide-field and confocal images of snail neurons (figure 5B). Fine axonal processes and detail on the cell soma can be distinguished in confocal mode but not in wide-field mode.

We also compared the results with those from a commercial CLSM. Figure 5C compares images taken of *cucurbita* with the DMD microscope and Leica SP2 CLSM. Although the contrast is better with the commercial system, the level of detail is similar. Four times line averaging was used for the CLSM therefore the overall speed of imaging was much slower than the DMD microscope. We also compared the axial resolution of both systems and as shown in figure 5D, the degree of axial sectioning for the DMD system was actually slightly better than the Leica system when comparing the same sized beads with objectives with similar magnification and the same numerical aperture.

### Calcium imaging

**Figure 6:**
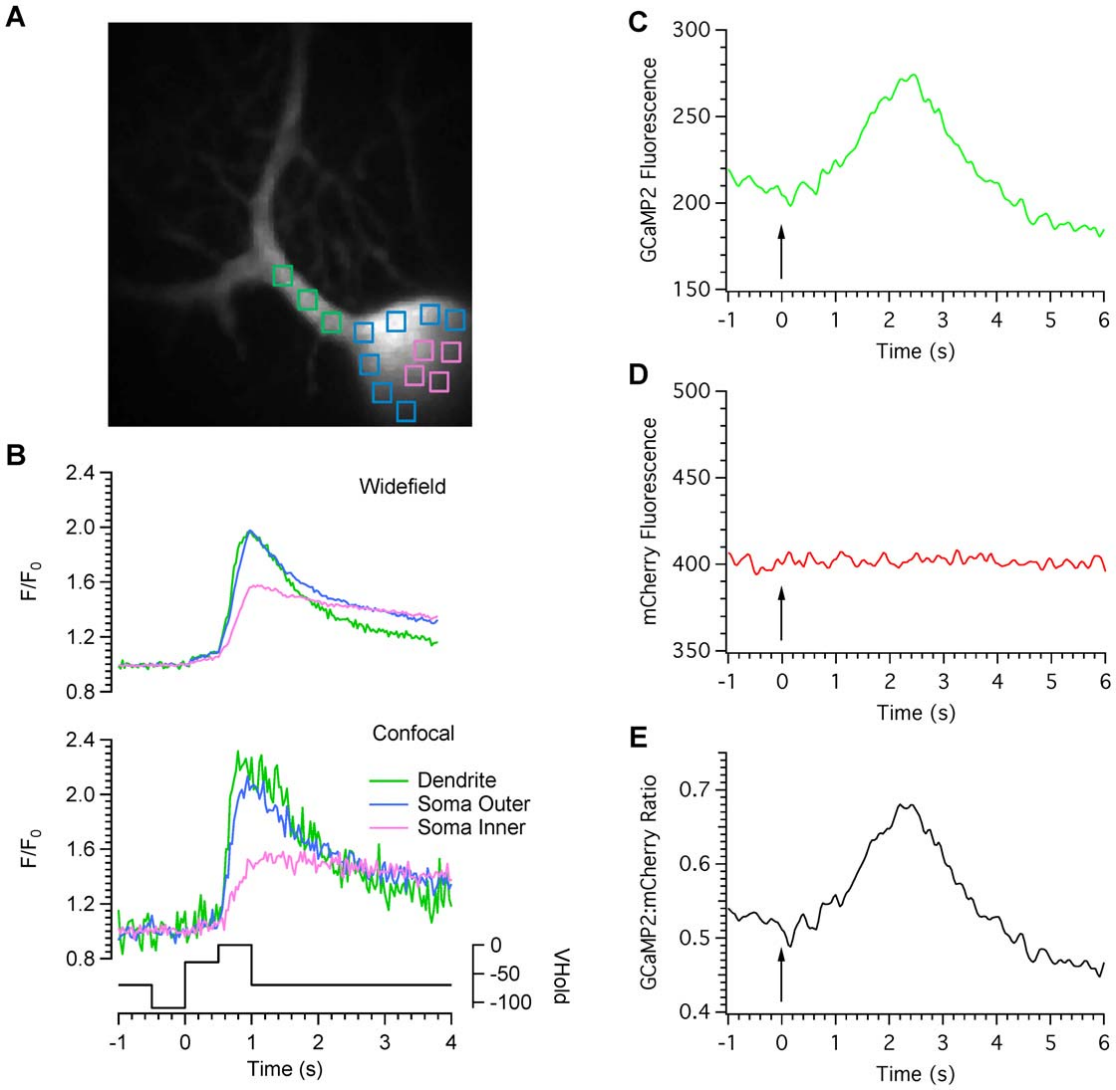
High-speed calcium imaging. A Purkinje cell was filled with the high affinity calcium indicator Oregon green BAPTA-1 and voltage clamped at −70 mV. Regions of interest were position over somatic and dendritic regions as shown in panel A. The cell was then pre-hyperpolarised to −110 mV, stepped to −40 mV and then to 0 mV to activate low- and high-voltage activated calcium channels respectively. Images were collected at 30 frames per second in wide-field (upper traces) and confocal (lower traces) modes. Note the differences in peak amplitude and time course for wide-field and confocal modes for each cellular compartment. The holding potential of the cell is shown under the calcium trace. C. Fluorescence measurements were taken from a hippocampal neurone transfected with a ratiometric, genetically encoded calcium indicator named GCaMP2-mCherry. Graphs C–E represent measurements of the GCaMP2, mCherry and ratio signals respectively. 180 pairs of images were collected at 40 ms intervals. The arrows indicate the onset of stimulation with 50 pulses at a rate of 20 Hz.

One of the main advantages of this type of microscope is the enhanced acquisition speed that accompanies scanning in parallel with multiple pinholes. Combined with an electron multiplying CCD camera, this leads to high quality confocal images acquired at speeds fast enough to capture biological events such as calcium transients in neurones. Figures 6 A and B provide an example of high-speed calcium imaging in a cerebellar Purkinje neuron filled with the high-affinity indicator Oregon Green Bapta-1. The Purkinje cell was voltage clamped and stepped from an initial holding potential of −70 mV, to −110 mV, then stepped to −40 mV and then to 0 mV to activate low voltage activated and high voltage activated calcium channels respectively. Images were collected at 30 frames per second in wide-field (upper traces) and confocal (lower traces) modes and measurements made from regions position within the soma, next to the somatic membrane and proximal dendrites. Although confocal measurements were more noisy than the wide-field measurements because of the vastly reduced amount of excitation light available, optical sectioning revealed a clear distinction between the kinetics of calcium mobilisation within each cellular compartment, consistent with previous reports of differential calcium signalling within somatic, perisomatic and dendritic compartments, previously revealed with confocal line-scanning techniques [16].

We next examined the suitability of this microscope for high-speed, ratiometric calcium imaging. A genetically encoded calcium indicator that consisted of GCaMP2 [14] fused to the red fluorescent protein mCherry was expressed in primary hippocampal neuronal cultures. GCaMP2 undergoes an increase in fluorescence on binding with calcium but mCherry does not. The ratio of GCaMP2:mCherry signals is therefore directly proportional to calcium concentration. Figure 6C–E illustrates fluorescent measurements of GCaMP2 (6C) and mCherry signals (6D) as well as the GCaMP2:mCherry ratio (6E) from a hippocampal neuron depolarised with a platinum bipolar electrode at a speed of 25 ratio pairs per second. The cell was activated 50 times at a rate of 50 Hz.

**Figure 7:**
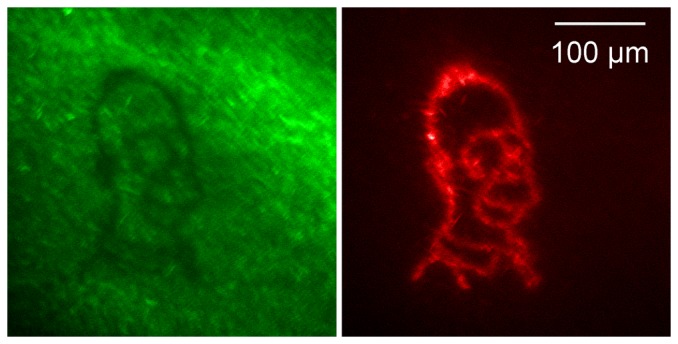
Photoactivation of mEOS2. A layer of bacteria expressing mEO2 was photo-activated with 405 nm light. The image on the left shows the resulting loss of green fluorescence where the photoactivation took place. The image on the right shows the appearance of red fluorescence.

As well as providing a means of optical sectioning, a spatial light modulator in the excitation path allows programmable illumination and photoactivation of photoactive material. A layer of bacteria expressing mEOS2 [15], a green fluorescent protein whose fluorescence emission changes to red on exposure to UV light was spread between a slide and a coverslip. A cartoon image was generated on the DMD and 405 nm light projected onto the specimen. As shown in figure 7, even with relatively low power LED light, the photoconversion of green to red fluorescence was readily apparent.

## Discussion

We have described a DMD based microscope that produces high-resolution confocal images at speeds limited by the frame rate of the camera. This microscope has several advantages over other, commercially available Nipkov disk type systems. First, it uses no macro moving parts and is freely programmable. Therefore, it is possible to modify the degree of optical sectioning by altering the size and spacing of pinholes. This flexibility allows easy optimisation for use with a range of objectives without having to change and realign a disk. The degree of sectioning can be altered from wide-field to a level of optical sectioning that is comparable to a commercial CLSM but at significantly higher speeds. The DMD can operate in a binary mode where the mirrors flip between on and off positions or in an 8-bit mode whereby the mirrors flicker at a frequency determined by the bit level. This allows the level of illumination within all or user-selected parts of the field of view to be programmed. Programmable illumination enables other modes of use including photolytic release of caged compounds of photoactivation of fluorescent proteins and fluorescent recovery after photobleaching (FRAP).

Our results indicate that three factors affect the image resolution; pinhole size, pinhole separation and illumination intensity. If the pinhole size is too large, axial resolution is reduced and image quality is impaired because the edges of the pinholes (mirrors) can be seen on the images. Shifting the centre of the pinhole position by a number of mirrors smaller than the size of the pinhole and repeating the entire scan can overcome this effect. This process does not need to have an impact on the speed of acquisition because the DMD can switch frames much faster than most cameras can sample. However, since the on-board memory of the ALP-3000 is limited, more frames per sequence means that fewer configurations can be loaded into memory at anyone time. The main influence on axial resolution is pinhole separation and a finite separation of at least 300 µm is required for a 60× objective. Although wider separations should produce better axial resolution, the concomitant exponential decline in excitation power limits the signal to noise ratio and this outweighs the theoretical improvement in axial resolution that increased pinhole separation should bring.

One of the main limitations of this and all other Nipkov disk type systems is the very inefficient use of excitation light. Because a reasonable level of confocality is only produced by wide separation of the pinholes most of the excitation light is discarded. Whereas this has been a limitation in the past, the development of more and more powerful light sources overcomes this problem. Whilst this is very costly for laser based systems, we have shown that our DMD microscope, using a low cost, multi-wavelength, LED based light system, compares very favourably with a commercial CLSM in terms of axial resolution and spatial resolution at up to 100 times the acquisition speed. This has significant implications for the cost of future confocal systems, particularly now that LEDs are powerful enough for photoactivation and uncaging.

Although the transmission of excitation light is wasteful, the emission pathway of this system is remarkably efficient. The use of a modified Offner triplet arrangement of mirrors to relay light from the DMD to the camera brings several advantages compared to a lens based system. First, this optical arrangement is important for preventing astigmatism of the image. The DMD does not behave like a single plain mirror because all of the micro mirrors lie in the same plane. Therefore, the wave front of the image formed on the DMD is tilted. A triplet mirror arrangement allows the wavefront to be positioned normal to the surface of the DMD and the use of mirrors rather than lenses minimises losses and improves efficiency of light collection. A second advantage is that the relay is achromatic and so provided the illumination of the specimen is even and achromatic, no wavelength-dependent alignment of images is necessary. Third, mirrors are much more efficient than lenses and so the detection efficiency of light emitted from the specimen is optimised.

### Areas for further development

Since this system was devised, higher resolution DMDs have been produced with smaller mirrors and an updated version of the ALP high-speed system has been developed with significantly larger amounts of on board memory and faster frame update times. Smaller mirrors are likely to allow improved image quality with cameras that have smaller pixels and by reducing the visibility of mirror edges that can be observed at some pinhole configurations. More memory allows a greater number of sequences to be stored in memory and faster DMD frame update speeds could allow better temporal resolution in combination with devices such as CMOS cameras that operate at speeds faster than CCD cameras.

Our current prototype lacks contrast compared to a commercial CLSM. The reason for this is interference by stray light. This can be removed by better engineering. Stray light is a large potential problem for this type of microscope because any pixel on the camera is only collecting light from the desired plane of focus for a fraction of the total exposure time. For example, if 64 DMD frames are required to scan the entire image, any one pixel on the camera is only collecting wanted light for 1/64^th^ of the exposure period. Any stray or unwanted background light will be collected for the entire exposure time. Therefore, it is essential to eliminate all sources of stray light to ensure the signal to noise ratio is as good as possible. An alternative approach is to replace the CCD with photo-sensitive devices that provide information about the time as well as the location of signals. If the time as well as the position from which the light originated is known, it is possible to discard unwanted stray light.

In conclusion, we have shown that this DMD-based microscope can achieve excellent levels of optical sectioning that compares well with a commercially available CLSM. An important advantage of the DMD microscope is speed. With our system, high-resolution images can be taken at speeds well above 30 frames per second and as little as 10 ms for bright specimens. The main advantage of this system is flexibility. The user can favour speed or high resolution, and it can be easily adapted to any kind of fluorescent microscope.
